# Evaluation of TikTok videos on acute pancreatitis: content quality and reliability analysis

**DOI:** 10.1186/s12889-024-18708-2

**Published:** 2024-05-02

**Authors:** Tianyang Mao, Xin Zhao, Kangyi Jiang, Jie Yang, Qingyun Xie, Jinqiang Fu, Bo Du, Zehua Lei, Fengwei Gao

**Affiliations:** 1 Department of Hepatopancreatobiliary Surgery, The People’s Hospital of Leshan, No.238, Baita Street, Leshan, 614000 China; 2 Diagnosis and Treatment Center for Liver, Gallbladder, Pancreas and Spleen System Diseases of Leshan, Leshan, China; 3 Liver Transplantation Center, State Key Laboratory of Biotherapy and Cancer Center, West China Hospital, Sichuan University and Collaborative Innovation Center of Biotherapy, Chengdu, China; 4 North Sichuan Medical College, Nanchong, China

**Keywords:** TikTok, Acute pancreatitis, Social media, Video platform, Public safety, DISCERN, Online health information, HONcode

## Abstract

**Background:**

Acute pancreatitis (AP) is a common acute digestive system disorder, with patients often turning to TikTok for AP-related information. However, the platform’s video quality on AP has not been thoroughly investigated.

**Objective:**

The main purpose of this study is to evaluate the quality of videos about AP on TikTok, and the secondary purpose is to study the related factors of video quality.

**Methods:**

This study involved retrieving AP-related videos from TikTok, determining, and analyzing them based on predefined inclusion and exclusion criteria. Relevant data were extracted and compiled for evaluation. Video quality was scored using the DISCERN instrument and the Health on the Net (HONcode) score, complemented by introducing the Acute Pancreatitis Content Score (APCS). Pearson correlation analysis was used to assess the correlation between video quality scores and user engagement metrics such as likes, comments, favorites, retweets, and video duration.

**Results:**

A total of 111 TikTok videos were included for analysis, and video publishers were composed of physicians (89.18%), news media organizations (13.51%), individual users (5.41%), and medical institutions (0.9%). The majority of videos focused on AP-related educational content (64.87%), followed by physicians’ diagnostic and treatment records (15.32%), and personal experiences (19.81%). The mean scores for DISCERN, HONcode, and APCS were 33.05 ± 7.87, 3.09 ± 0.93, and 1.86 ± 1.30, respectively. The highest video scores were those posted by physicians (35.17 ± 7.02 for DISCERN, 3.31 ± 0.56 for HONcode, and 1.94 ± 1.34 for APCS, respectively). According to the APCS, the main contents focused on etiology (*n* = 55, 49.5%) and clinical presentations (*n* = 36, 32.4%), followed by treatment (*n* = 24, 21.6%), severity (*n* = 20, 18.0%), prevention (*n* = 19, 17.1%), pathophysiology (*n* = 17, 15.3%), definitions (*n* = 13, 11.7%), examinations (*n* = 10, 9%), and other related content. There was no correlation between the scores of the three evaluation tools and the number of followers, likes, comments, favorites, and retweets of the video. However, DISCERN (*r* = 0.309) and APCS (*r* = 0.407) showed a significant positive correlation with video duration, while HONcode showed no correlation with the duration of the video.

**Conclusions:**

The general quality of TikTok videos related to AP is poor; however, the content posted by medical professionals shows relatively higher quality, predominantly focusing on clinical presentations and etiologies. There is a discernible correlation between video duration and quality ratings, indicating that a combined approach incorporating the guideline can comprehensively evaluate AP-related content on TikTok.

## Introduction

Acute pancreatitis (AP) represents a prevalent acute abdomen condition in the gastrointestinal system, characterized by a cascade of pathological changes, including tissue self-digestion, edema, effusion, and even necrosis, infection of the pancreas and its adjacent tissues [1, 2]. These changes are caused by abnormal activation of pancreatic enzymes due to multifarious etiologies. Most patients have mild acute pancreatitis, which is self-resolving and has a good prognosis. However, approximately 20% of patients will progress to moderate or severe acute pancreatitis, often accompanied by multiple organ failure or systemic inflammation, culminating in a mortality rate of 20-40% [3].

With the development of the information age, many health-related video content have appeared in social media software, positioning these platforms as important sources for public health information acquisition. TikTok, in particular, is one of the world’s most populated short-video social platforms and plays a significant role in transmitting disease-related health information [4, 5].

Studies have shown that TikTok has great potential for health information dissemination during public safety and health crises, such as COVID-19 pandemic and monkeypox endemic [6–8]. Furthermore, healthcare professionals can also release disease-related educational content via TikTok, facilitating the spread of scientific knowledge to the general public. However, due to the low barriers for TikTok user registration and video posting, individuals without medical expertise, in addition to medical professionals, can also post relevant videos. While inclusive, this democratization of content creation concerns the video quality and reliability of the health information presented. Some early studies evaluated the quality and reliability of disease-related videos such as gallstones, liver cancer, and diabetes on TikTok, but the results were unsatisfactory [9–11].

Conversely, the content, quality, and reliability of AP-related videos on TikTok remain unclear. For this reason, the present study employed two evaluation instruments - DISCERN and HONcode to analyze the AP-related videos on TikTok. Additionally, an AP Content Score(APCS) was incorporated as a supplementary evaluation for the videos [12–14] to comprehensively evaluate the quality and reliability of AP-related content on TikTok, and to determine whether the platform provides the public with accurate AP-related information.

## Methods

### Ethical considerations

This study did not involve the use of clinical data, human specimens, or laboratory animals. All information was sourced from publicly available TikTok videos, and none of the data has personal privacy implications. In addition, the present study entailed no interaction with users and, therefore, does not require ethical review.

### Search strategy and data collection

**Fig. 1:**
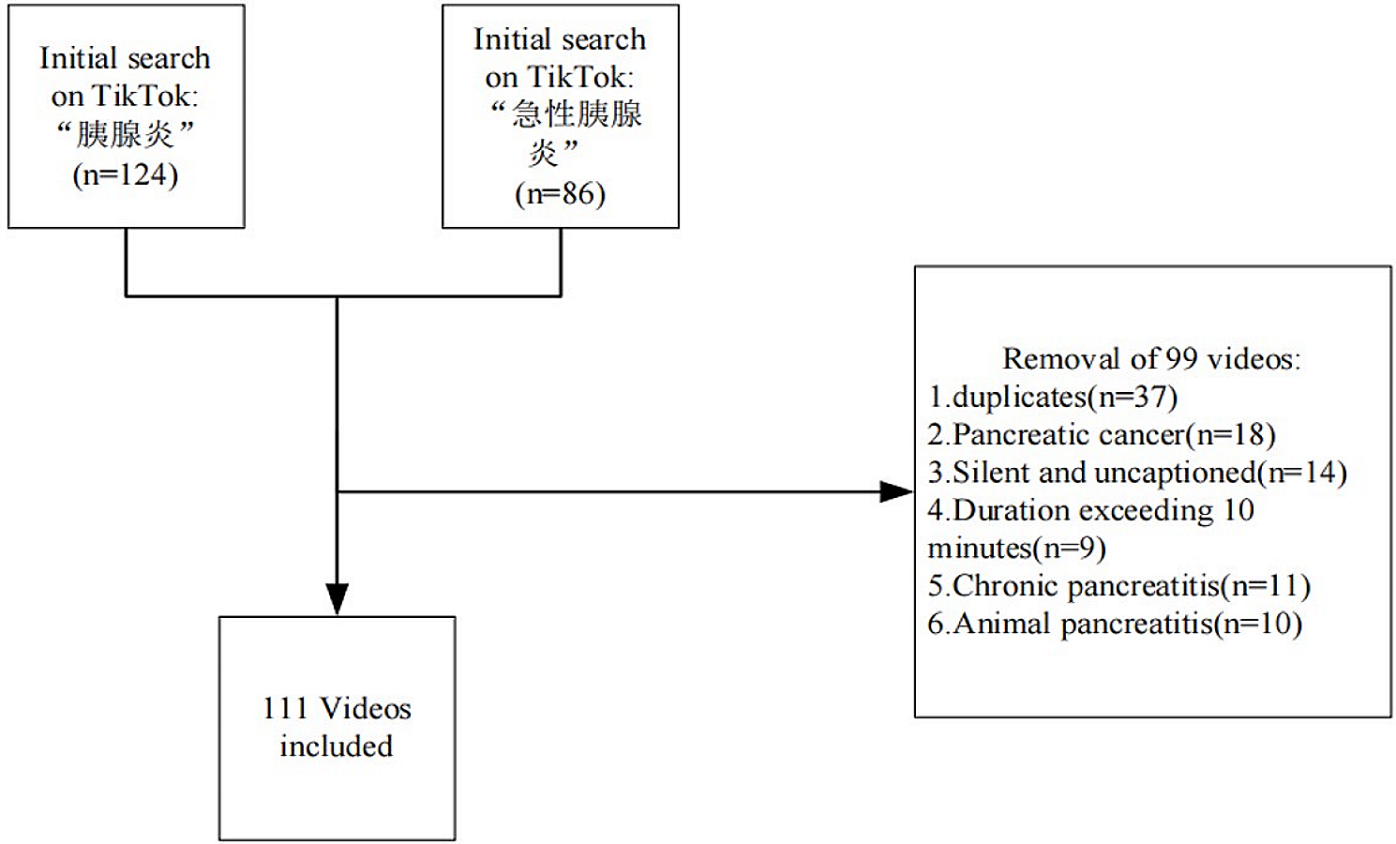
Flowchart for video retrieval and selection

A new TikTok account was registered, and a search was conducted using keywords such as “胰腺炎” or “急性胰腺炎” (“Pancreatitis” and “Acute Pancreatitis” in Chinese, respectively). The cutoff date for video retrieval was set to September 20, 2023, yielding 210 relevant videos. Since the inclusion of videos was comprehensive, there was no bias caused by historical records. After a thorough review, videos were excluded based on the following criteria: (1) duration exceeding 10 min, (2) duplicates, (3) chronic pancreatitis-related, (4) silent and uncaptioned, (5) on animal pancreatitis, and (6) pancreatic cancer-related videos. Ultimately, 111 videos were deemed suitable for inclusion in the analysis (Fig. 1).

All relevant videos were downloaded and systematically cataloged through numerical identification. We extracted and recorded the information of each video, including metrics such as the number of followers, likes, comments, favorites, retweets, duration, publisher identity, and content classification. These data were methodically documented in an Excel spreadsheet.

We categorized the videos based on their source into four groups and based on their content into three groups. Video sources are classified as follows: (1) medical institutions, (2) news media organizations, (3) physician users, and (4) individual users. The video content is classified as follows: (1) AP-related educational content, (2) physicians’ diagnostic and treatment records, and (3) personal experiences. Videos for professionals are further classified as follows: (1) physicians that manage/encounter AP, (2) those who do not, and (3) other medical users or practitioners of Traditional Chinese Medicine whose specific expertise was unknown.

### Video evaluation

DISCERN, a reliable tool for assessing the quality of health information, was initially designed to assess the quality of written information regarding treatment options [13]. It has been widely used to evaluate the quality of video information [15–17]. It consists of 16 questions divided into three sections to assess the reliability of the information, treatment, and overall evaluation. Each question is scored on a scale from 1 to 5 points, with ‘No’ scoring 1 point, ‘Partially’ 3 points, and ‘Yes’ 5 points. The sum of points from all 16 questions constitutes the total score. A total score of ≤ 26 indicates very poor quality, 27–38 poor, 39–50 average, 51–61 good, and a score of ≥ 62 is indicative of excellent quality [9].

The HONcode is a tool designed to harmonize and standardize the quality of online health information. It has eight principles: authority, complementarity, privacy, attribution, justifiability, transparency, financial disclosure, and advertising policy [14]. Each principle is assessed on a numeric scale, with 1 point for each question; a score ranging from 0 to 2 indicates low quality, 3 to 5 indicates average quality, and a score between 6 and 8 denotes high quality [18].

APCS is an evaluative tool we have developed according to the guidelines for the diagnosis and treatment of acute pancreatitis [12, 19]. It serves as a supplementary instrument to the DISCERN and HONcode tools. It contains 14 aspects: disease definition, etiology, clinical presentation, diagnosis, examination, pathophysiology, severity classification, AP manifestations in childhood, during pregnancy, and in the elderly, as well as treatment, prevention, complications, and sequelae. Relevant content mentioned earns 1 point per dimension. A score of 0 to 4 indicates low content, 5 to 9 indicates average, and a score ranging from 10 to 14 denotes comprehensive content (Table 1).

**Table 1:** AP content score

Does it mention the following?	Score	
	Yes	No
1. Disease Definition	1	0
2. Etiology	1	0
3. Clinical Presentation	1	0
4. Diagnosis	1	0
5. Examination	1	0
6. Pathophysiology	1	0
7. Severity Classification	1	0
8. AP Manifestations In Childhood	1	0
9. AP Manifestations During Pregnancy	1	0
10. AP In The Elderly	1	0
11. Treatment	1	0
12. Prevention	1	0
13. Complications	1	0
14. Sequelae	1	0
Scores: Low content: 0–4; Average: 5–9; Comprehensive content: 10–14		

AP: Acute Pancreatitis

Each video was assessed independently by two evaluators using the three tools mentioned above. In instances of a discrepancy between the two evaluators’ scores, all group members convened to discuss, reaching a consensus.

### Statistical analysis

SPSS version 26.0 (IBM Corporation) was used for statistical analysis. For measurement data adhering to a normal distribution, it is expressed as mean ± standard deviation. A T-test was used for inter-group comparison. When matching the skew distribution, data are expressed as the median (interquartile distance), and the Mann-Whitney U test was used for comparison between groups. The Kruskal-Wallis test was employed to compare multiple data sets for non-normally distributed quantitative variables. Two sets of continuous numerical data were analyzed using Pearson correlation analysis. The count data were expressed as the number of cases (percentage) [n(%)]. The comparison between groups was performed by the Chi-square (X^2^) test. A *p*-value of < 0.05 was considered indicative of statistical significance.

## Results

### Classification of videos

The total number views of videos retrieved related to the specified topic reached 358 million times. After the exclusion process of non-relevant videos, 111 videos were included. Most of the videos were posted by physicians (*n* = 89, 80.18%), followed by news media organizations (*n* = 15, 13.51%), non-professional individual users (*n* = 6, 5.41%), and medical institutions (*n* = 1, 0.90%). Physician users were further categorized into manage/encounter AP (*n* = 62, 69.66%), those who do not (*n* = 12, 13.48%), and other unspecified specializations or related to Traditional Chinese Medicine (*n* = 15, 16.86%). Regarding video content, most of the videos are AP-related educational content (*n* = 72,64.87%). The remaining content consisted of physicians’ records during diagnosis and treatment (*n* = 17, 15.32%) and personal experiences (*n* = 22, 19.81%) (Table 2). Videos produced by news media organizations gained higher engagement metrics, including followers, likes, comments, favorites, and retweets, compared to those posted by physicians and non-professional individual users. Moreover, videos posted by physicians tended to be longer in duration, showing significant differences (Table 3).

**Table 2:** Characterization of videos

Overall videos, *n*(%)	111(100)
Publisher identity, n(%)	
Physicians	89(80.18)
News media organizations	15(13.51)
Individuals	6(5.41)
Medical institutions	1(0.90)
Doctor category, n(%)	
manage/encounter AP	62(69.66)
those who do not	12(13.48)
Other/TCM	15(16.86)
Video content, n(%)	
Educational content	72(64.87)
Records	17(15.32)
Experience	22(19.81)
Fans(w)	5.30(1.05,61.75)
Likes	934(330,3983.50)
Comments	116(31,548.50)
Favorites	220(81.50,683)
Retweets	359(114.50,2493)
Duration	45(31,82)
DISCERN	34(26,38)
HONcode	3(3,4)
APCS	2(1,2)

TCM: Traditional Chinese medicine; APCS: Acute Pancreatitis Content Score

**Table 3:** Characteristics of the videos in publisher identity

Characteristics (*N* = 110)	Physicians (*n* = 89), median (IQR)	News media(*n* = 15), median(IQR)	Individuals(*n* = 6), median(IQR)	*p*
Fans(1k)	4.3(1,39.80)	326.20(188.10,741.90)	0.50(0.30,73)	< 0.001
Likes	804(322,2702)	3758(2215,8093.50)	591.50(270,7720)	0.048
Comments	91(26,447)	391(232,3096)	531(192,1066)	0.01
Favorites	191(79,668)	406(193,1072.50)	63.50(44,400)	0.341
Retweets	336(110,2115)	1935(614,26500)	130.50(64,199)	0.023
Duration	48(36,83)	25(10,40)	33.50(22,41)	0.007
DISCERN	34(31,40)	23(21,25)	22(21,25)	< 0.001
HONcode	3(3,4)	3(2,4)	0(0,0)	< 0.001
APCS	2(1,2)	2(1,2.50)	0.50(0,1)	0.012

Since only 1 video was published by a medical institution, it was automatically excluded from analysis. APCS: Acute Pancreatitis Content Score;1k: one thuosand

### Video quality evaluation using DISCERN and HONcode

**Fig. 2:**
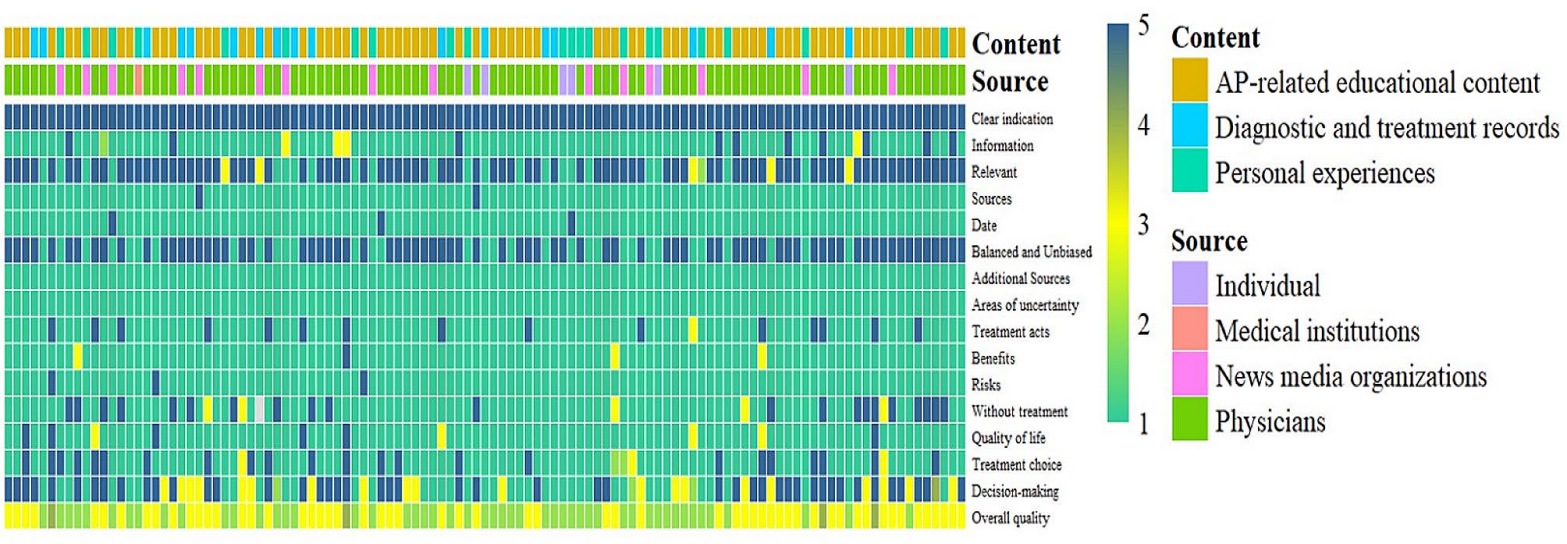
DISCERN score for videos. Use heatmap to represent DISCERN scores. Rows represent rated items, columns represent individual videos (*n* = 111). The video category is shown in the top row of the heatmap. AP: acute pancreatitis

The mean DISCERN score for the 111 videos was 33.05 ± 7.87, categorizing the overall quality score as poor (Fig. 2). Specifically, 25.23% (*n* = 28) of the videos were rated very poor, 53.15%(*n* = 59) poor, 18.02% (*n* = 20) fair, and 3.60% (*n* = 4) good, with none achieving an excellent rating (Table 4). Regarding video publisher identity, those posted by physicians (median 34; range 31 to 40) scored significantly higher compared to those posted by news media (median 23; range 21 to 25) and non-professional individual users (median 22; range 21 to 25) (*P* < 0.001, Table 3). Moreover, regarding content, videos centered on AP-related knowledge dissemination (median 36; range 34 to 42) received higher scores compared to diagnosis and treatment records (median 29; range 22 to 34) and personal experience (median 24; range 21 to 26), with these variations being statistically significant (*P* < 0.001, Table 5).

**Table 4:** Analyze the video according to its rating

Scores	Value, *n*(%)
DISCERN	
≤ 26(very poor)	28(25.23)
27–38(poor)	59(53.15)
39–50(fair)	20(18.02)
51–61(good)	4(3.60)
≥ 62(excellent)	0
HONcode	
0–2(low quality)	12(10.81)
3–5(general quality)	99(89.19)
6–8(high quality)	0
APCS	
0–4(less content)	107(96.40)
5–8(general content)	4(3.60)
9–14(rich content)	0

APCS: Acute Pancreatitis Content Score

**Table 5:** Based on the content of the video analysis

Evaluation tools	Educational content (*n* = 72), median (IQR)	Record(*n* = 17), median (IQR)	Experience(*n* = 22), median (IQR)	*p*
DISCERN	36(34,42)	29(22,34)	24(21,26)	< 0.001
HONcode	3(3,3)	4(3,4)	3(2,4)	0.008
APCS	2(1,3)	1(1,1)	1(1,3)	0.002

APCS: Acute Pancreatitis Content Score

The mean HONcode score was 3.09 ± 0.93, indicating an overall quality as general. Most videos were of general quality (*n* = 99, 89.19%), with none achieving a high-quality rating (Table 4). Concerning the video publisher identity, physicians (median 3; range 3 to 4) and news media (median 3; range 2 to 4) had comparable scores; however, non-professional users scored significantly lower with a median of 0 (*P* < 0.001, Table 2). In terms of content, recorded videos of medical students’ diagnosis and treatment processes (median 4; range 3 to 4) scored higher than those on AP-related knowledge dissemination (median 3; range 3 to 3) and personal experiences (median 3; range 2 to 4), with the difference being statistically significant (*P* = 0.008, Table 5).

The mean APCS score was 1.86 ± 1.30, suggesting that the video content minimally covered relevant content, mainly related to the AP clinical manifestation (*n* = 36, 32.40%) and etiology (*n* = 55, 49.5%), followed by the AP treatment (*n* = 24, 21.60%), severity (*n* = 20, 18%), and prevention (*n* = 19, 17.10%). Other aspects, like pathophysiology (*n* = 17, 15.3%), definition (*n* = 13, 11.70%), and examination (*n* = 10, 9%) were less frequently mentioned. Videos rarely addressed diagnosis (*n* = 4, 3.60%), complications (*n* = 4, 3.60%), AP during pregnancy (*n* = 2, 1.80%), AP in children (*n* = 1, 0.90%), sequelae (*n* = 1, 0.90%), and AP in the elderly (*n* = 0, 0%). When comparing the identity of video publishers, physicians and news media had similar median scores (median 2; range 1 to 2), while non-professional individual users provided notably less coverage (median 0.5; range 0 to 1) (Table 3). Regarding content, videos focusing on AP-related knowledge dissemination (median 2; range 1 to 3) addressed significantly more guide-relevant content (*P* = 0.002, Table 5).

### Correlation analysis

The analysis revealed a moderate correlation between the number of fans and likes (*r* = 0.469, *P* < 0.001), favorites (*r* = 0.482, *P* < 0.001), and retweets (*r* = 0.418, *P* < 0.001). A strong correlation was observed between likes and comments (*r* = 0.627, *P* < 0.001), favorites (*r* = 0.813, *P* < 0.001), and retweets (*r* = 0.832, *P* < 0.001). Additionally, comments showed a strong correlation with favorites (*r* = 0.475, *P* < 0.001) and retweets (*r* = 0.604, *P* < 0.001). There was a strong correlation between favorites and retweets (*r* = 0.680, *P* < 0.001). However, no correlation was found between video duration and other variables. The DISCERN score was moderately correlated with video duration (*r* = 0.309, *P* = 0.001). There was no observed correlation between the HONcode score and other variables. Finally, APCS was significantly correlated with the DISCERN (*r* = 0.407, *P* < 0.001) and HONcode scores (*r* = 0.449, *P* < 0.001), suggesting a correlation between these evaluation metrics (Table 6).

**Table 6:** Pearson correlation analysis between data

	Fans(w)	Likes	Comments	Favorites	Retweets	Duration	Honcode	DISCERN	APCS
Fans(w)	1								
Likes, r/p	0.469**/<0.001	1							
Comments, r/p	0.180/0.059	0.627**/<0.001	1						
Favorites, r/p	0.482**/0.001	0.813**/<0.001	0.475**/<0.001	1					
Retweets, r/p	0.418**/<0.001	0.832**/<0.001	0.604**/<0.001	0.680**/<0.001	1				
Duration, r/p	-0.068/0.480	0.139/0.146	0.038/0.689	0.089/0.354	-0.024/0.803	1			
HONcode, r/p	-0.111/0.245	-0.017/0.860	-0.227*/0.017	-0.070/0.466	-0.015/0.879	0.045/0.637	1		
DISCERN, r/p	-0.218*/0.021	-0.073/0.446	-0.162/0.090	0.039/0.688	-0.119/0.212	0.309**/0.001	0.142/0.137	1	
APCS, r/p	0.080/0.407	0.087/0.365	-0.049/0.606	0.188*/0.048	0.107/0.263	0.407**/<0.001	0.086/0.369	0.449**/<0.001	1

APCS: Acute Pancreatitis Content Score

**: At level 0.01 (two-tailed), the correlation was significant

## Discussion

A study showed that 72% of the public uses at least one social media platform [5], underscoring its significant role in daily life. TikTok, in particular, is one of the most representative platforms. The ability of TikTok to spread information is very powerful and has been notably demonstrated during the COVID-19 pandemic [20]. With TikTok’s rising popularity, more medical professionals are leveraging the platform to share their expertise, facilitating patients’ access to medical information. Acute pancreatitis (AP), a common acute abdominal condition of the digestive system, may prompt patients to seek information on TikTok before medical treatment. To date, no studies have assessed the accuracy of AP-related information available on TikTok. This gap is crucial because incorrect or low-quality video content can lead to delayed patient visits and misdiagnoses.

The overall DISCERN score observed in this study was low, aligning with previous findings [21, 22]. Among the 111 included videos, 87 (78.38%) were rated as poor or below, comprising the majority of the videos. We believe this may be partially attributed to TikTok’s emphasis on short video content. Correlation analysis showed a significant positive correlation between DISCERN scores and video duration, corroborating with the results of Sun and colleagues [9]. The average duration of all the videos in this study was (62.84 ± 52.97) seconds, which may have contributed to lower scores due to the small amount of information conveyed in the limited time. It is worth noting that DISCERN was initially designed to evaluate the quality of treatment-related information. Its second section comprises six questions about “treatment”, which can result in notably reduced DISCERN scores if the video lacks content on AP treatment. However, most studies evaluating video quality have used DISCERN as an evaluation tool [21, 22], which was incomplete. This issue remains unsolved.

Employing authoritative guidelines to evaluate the video quality is considered a scientifically robust approach. The APCS includes the content mentioned in the AP guidelines and provides a comprehensive assessment of the amount of video content coverage. Due to the length of time, the video content mainly talks about the clinical presentations and causes of AP, and a few talk about the treatment, severity, prevention and pathophysiology of AP, which may be more concerned by the general public. The mean APCS score was 1.86 ± 1.30, with videos produced by medical professionals and news media reporting mainly professional knowledge. In contrast, non-professional users mainly reported their own experiences, which contributed minimally to the dissemination of disease-related knowledge.

The HONcode evaluates video quality from various aspects and is a criterion for internet information. Its score appears to be relatively independent of the videos’s specific content. Correlation analysis indicated no correlation between HONcode score and video duration. The professionalism of AP videos on TikTok is notable, with 92 (82.9%) of the videos narrated by professionals, mainly addressing the relationship between healthcare providers and patients. However, the other six principles are rarely satisfied, culminating in an overall average quality level, aligning with the findings of Goobie and colleagues [23] et al. This result may be attributed to TikTok’s low barriers to account registration and video uploading, as the platform has not set these norms as a requirement for video posting. In addition, an examination of all videos revealed a lack of cited references for the content mentioned, which is one of the essential reasons influencing video quality and reliability [24].

The three evaluation tools employed in this study reveal that videos posted by medical professionals achieve the highest scores; however, they garner the least number of followers, likes, comments, favorites, and retweets. This discrepancy indicates that relatively high-quality videos do not attract proportionate attention. A significant positive correlation among these tools suggests they reflect the popularity of videos to some extent [25]. News media platforms and individual users tend to upload content that is popular with the general audience, often compromising the video quality. In contrast, medical professionals prioritize disseminating disease-related knowledge. The general public may prefer to watch popular videos, and TikTok cannot guide viewers toward more informative and high-quality content.

There are some limitations in this study. Firstly, as the TikTok videos analyzed are exclusively in Chinese, the applicability of these findings to other countries remains uncertain. The video quality needs further evaluation from widely used social media, such as Youtube and Facebook, across many countries. Secondly, in this study, we found that DISCERN could not comprehensively evaluate video quality, which led us to develop the APCS as a supplement for DISCERN. However, establishing an official, comprehensive evaluation tool for content quality evaluation remains necessary for future research. Lastly, the issue of duration relevance must be considered. While this study indicates the current inadequacy of video quality on TikTok, it cannot predict the future emergence of higher-quality AP-related videos on the platform.

## Conclusion

The overall quality of AP-related videos posted on TikTok is generally poor. However, content uploaded by medical professionals demonstrates relatively high quality, predominantly focusing on clinical manifestations and etiology. Notably, a certain correlation exists between a video’s duration and quality rating. Combining guidelines into the evaluation process facilitates a more comprehensive assessment of the quality of AP-related content on TikTok.

## Data Availability

Data are available upon reasonable request. Please contact Tianyang Mao, Email: tianyangmao@126.com.

## References

[1] Petrov MS, Yadav D (2019). Global epidemiology and holistic prevention of pancreatitis. Nat Rev Gastroenterol Hepatol.

[2] Lee PJ, Papachristou GI (2019). New insights into acute pancreatitis. Nat Rev Gastroenterol Hepatol.

[3] Schepers NJ, Bakker OJ, Besselink MG, Ahmed Ali U, Bollen TL, Gooszen HG (2019). Impact of characteristics of organ failure and infected necrosis on mortality in necrotising pancreatitis. Gut.

[4] Baumann E, Czerwinski F, Rosset M, Seelig M, Suhr R (2020). [How do people in Germany seek health information? Insights from the first wave of HINTS Germany]. Bundesgesundheitsblatt Gesundheitsforschung Gesundheitsschutz.

[5] Comp G, Dyer S, Gottlieb M. Is TikTok the next social media frontier for medicine? AEM Educ Train. 2021;5. 10.1002/aet2.10532 10.1002/aet2.10532 PMC8155692 34095694

[6] Southwick L, Guntuku SC, Klinger EV, Seltzer E, McCalpin HJ, Merchant RM (2021). Characterizing COVID-19 content posted to TikTok: public sentiment and response during the First Phase of the COVID-19 pandemic. J Adolesc Health.

[7] Shi A, El Haddad J, Cai P, Song S, Wang YJ, Liu Q, et al. Mpox (monkeypox) information on TikTok: analysis of quality and audience engagement. BMJ Glob Health. 2023;8. 10.1136/bmjgh-2022-011138 10.1136/bmjgh-2022-011138 PMC10016284 36918216

[8] Zhu C, Xu X, Zhang W, Chen J, Evans R. How health communication via Tik Tok makes a difference: a content analysis of Tik Tok accounts run by Chinese provincial health committees. Int J Environ Res Public Health. 2019;17. 10.3390/ijerph17010192 10.3390/ijerph17010192 PMC6981526 31892122

[9] Sun F, Zheng S, Wu J (2023). Quality of information in gallstone disease videos on TikTok: cross-sectional study. J Med Internet Res.

[10] Zheng S, Tong X, Wan D, Hu C, Hu Q, Ke Q (2023). Quality and reliability of Liver Cancer-related short Chinese videos on TikTok and Bilibili: cross-sectional content analysis study. J Med Internet Res.

[11] Kong W, Song S, Zhao YC, Zhu Q, Sha L (2021). TikTok as a Health Information source: Assessment of the quality of information in diabetes-related videos. J Med Internet Res.

[12] Szatmary P, Grammatikopoulos T, Cai W, Huang W, Mukherjee R, Halloran C (2022). Acute Pancreatitis: diagnosis and treatment. Drugs.

[13] Charnock D, Shepperd S, Needham G, Gann R (1999). DISCERN: an instrument for judging the quality of written consumer health information on treatment choices. J Epidemiol Community Health.

[14] Boyer C, Selby M, Scherrer JR, Appel RD (1998). The Health on the Net Code of Conduct for medical and health websites. Comput Biol Med.

[15] Chen Z, Pan S, Zuo S (2022). TikTok and YouTube as sources of information on anal fissure: a comparative analysis. Front Public Health.

[16] Babar M, Loloi J, Patel RD, Singh S, Azhar U, Maria P (2022). Cross-sectional and comparative analysis of videos on erectile dysfunction treatment on YouTube and TikTok. Andrologia.

[17] Du RC, Zhang Y, Wang MH, Lu NH, Hu Y (2023). TikTok and Bilibili as sources of information on Helicobacter pylori in China: A content and quality analysis. Helicobacter.

[18] Wilkens FM, Ganter C, Kriegsmann K, Wilkens H, Kahn N, Goobie GC (2022). YouTube-videos for patient education in lymphangioleiomyomatosis?. Respir Res.

[19] Mederos MA, Reber HA, Girgis MD (2021). Acute Pancreatitis: a review. JAMA.

[20] Gottlieb M, Dyer S (2020). Information and disinformation: Social Media in the COVID-19 Crisis. Acad Emerg Med.

[21] Meade MJ, Meade EA, Dreyer CW (2022). Orthodontic clear aligners and TikTok videos: a content, reliability and quality analysis. Int Orthod.

[22] Lahooti A, Hassan A, Critelli B, Westerveld D, Newberry C, Kumar S (2023). Quality and Popularity trends of Weight loss Procedure videos on TikTok. Obes Surg.

[23] Goobie GC, Guler SA, Johannson KA, Fisher JH, Ryerson CJ (2019). YouTube Videos as a source of misinformation on idiopathic pulmonary fibrosis. Ann Am Thorac Soc.

[24] Xue X, Yang X, Xu W, Liu G, Xie Y, Ji Z (2022). TikTok as an information hodgepodge: evaluation of the quality and reliability of Genitourinary Cancers Related Content. Front Oncol.

[25] Yu JS, Carr JB, Thomas J, Kostas J, Wang Z, Khilnani T (2021). Trends in Patient, Physician, and Public Perception of Ulnar Collateral Ligament Reconstruction Using Social Media Analytics. Orthop J Sports Med.

